# In and out: Traffic and dynamics of thrombopoietin receptor

**DOI:** 10.1111/jcmm.16878

**Published:** 2021-08-27

**Authors:** Anita Roy, Saurabh Shrivastva, Saadia Naseer

**Affiliations:** ^1^ Kusuma School of Biological Sciences Indian Institute of Technology New Delhi India

**Keywords:** JAK2, myeloproliferation, receptor dimerization, thrombopoietin receptor, traffic

## Abstract

Thrombopoiesis had long been a challenging area of study due to the rarity of megakaryocyte precursors in the bone marrow and the incomplete understanding of its regulatory cytokines. A breakthrough was achieved in the early 1990s with the discovery of the thrombopoietin receptor (TpoR) and its ligand thrombopoietin (TPO). This accelerated research in thrombopoiesis, including the uncovering of the molecular basis of myeloproliferative neoplasms (MPN) and the advent of drugs to treat thrombocytopenic purpura. TpoR mutations affecting its membrane dynamics or transport were increasingly associated with pathologies such as MPN and thrombocytosis. It also became apparent that TpoR affected hematopoietic stem cell (HSC) quiescence while priming hematopoietic stem cells (HSCs) towards the megakaryocyte lineage. Thorough knowledge of TpoR surface localization, dimerization, dynamics and stability is therefore crucial to understanding thrombopoiesis and related pathologies. In this review, we will discuss the mechanisms of TpoR traffic. We will focus on the recent progress in TpoR membrane dynamics and highlight the areas that remain unexplored.

## 1 INTRODUCTION

**FIGURE 1:**
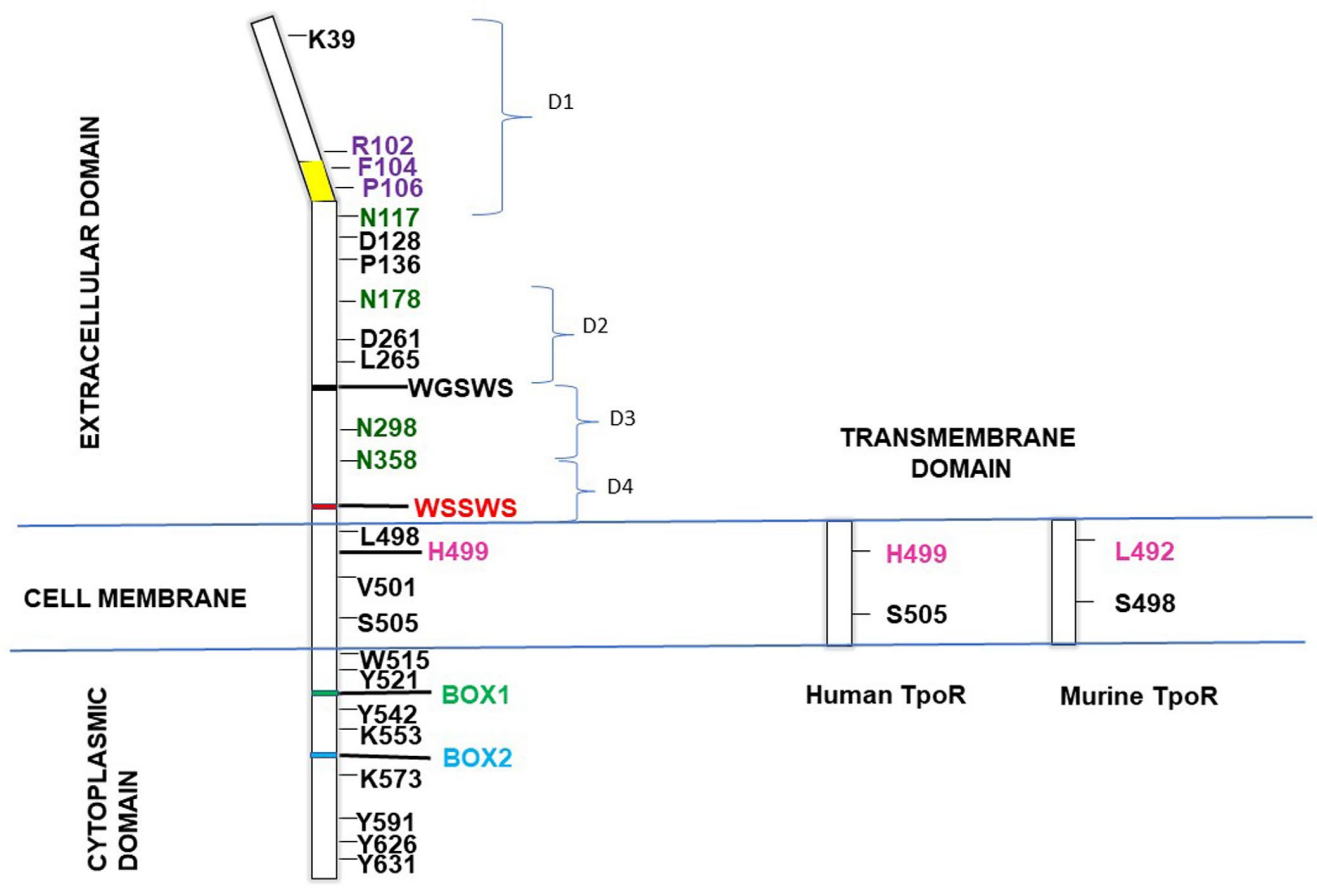
Schematic TpoR structure depicting the position of the important residues. The conserved WSSWS motifs (shown in red) in the extracellular domain of receptor and Box 1 (green) and Box 2 (blue) part of the cytosolic domain are indicated. Four N‐glycosylation N117, N178, N298 and N358 (in green) and residues mutated in hereditary thrombocytosis R102, F104 and P106 (in violet) are shown. The hydrophobic patch in the TpoR extracellular domain is indicated in yellow. Eltrombopag‐binding site at residue H499 of human TpoR is indicated in magenta. Human H499 and its equivalent murine L492 are shown along with the position S505 (human) and S498 (murine) for comparison

Thrombopoietin receptor (TpoR), also called c‐MPL (cellular‐myeloproliferative leukaemia), is a cytokine receptor found on haemangioblasts, hematopoietic stem cells (HSCs), megakaryocytes and platelets.^1^, ^2^ TpoR was first discovered in 1992 by Vigon et al. in the prospect of finding the human homolog of murine v‐mpl that was known to cause an acute myeloproliferative syndrome in mice.^3^, ^4^ Subsequently, the ligand for TpoR called thrombopoietin (TPO) was cloned by several groups in 1994.^5^, ^6^, ^7^, ^8^, ^9^, ^10^ Among them, Wendling et al.^10^ utilized post‐irradiated (cytopenic) mouse sera and observed selective growth of TpoR‐expressing cells. Interestingly, an increase in serum TPO has also been detected in *c*‐*mpl* ^−/−^ mice.^11^, ^12^, ^13^, ^14^ TPO is involved in the process of self‐renewal of HSCs and the production of platelets.^11^, ^12^, ^13^, ^15^, ^16^, ^17^ TPO binds with high efficiency to TpoR on the platelet surface, leading to the destruction of TPO by platelets. This, in turn, regulates plasma TPO levels. Interestingly, hepatocyte‐specific knock‐out of *Thpo* resulted in decreased platelet counts, raising the possibility for a cross‐organ regulation of the production of TPO by circulating platelets.^18^ Circulating plasma TPO drives megakaryopoiesis, thereby regulating platelet counts.^19^, ^20^ TpoR is structurally similar to members of the class I cytokine receptor superfamily, which includes erythropoietin receptor (EpoR), growth hormone receptor (GHR), granulocyte colony‐stimulating factor receptor (G‐CSFR) and granulocyte megakaryocyte colony‐stimulating factor receptor (GM‐CSFR). TpoR consists of 635 amino acids and can be divided into three functional regions: extracellular domain, transmembrane domain and cytoplasmic domain (Figure 1).

The extracellular domain exhibits a size of 466 aa and is nearly double the size of the extracellular domains of the homologous EpoR and GHR. This is due to the presence of two cytokine receptor modules (CRM‐1 and CRM‐2). The extracellular domain essentially constitutes a sensor. Each of the CRMs is composed of a pair of fibronectin‐III (FNIII)‐like domains (D1 and D2 in CRM‐1 and D3 and D4 in CRM‐2) and two pairs of cysteines. A conserved WSXWS box characteristic of type I receptors is present at the membrane‐proximal end of CRM‐2.^21^ The fibronectin‐III‐like domain present in each CRM is composed of 7 antiparallel β strands and is inter‐connected by the hinge region.^21^, ^22^ D261 and L265 residues^23^ within CRM‐1 (D2) are the primary site for TPO binding,^24^ and D128 and P136 residues between D1 and D2 maintain the conformation of ligand‐binding elbow.^25^ Hence, deletion of CRM‐1 results in loss of TPO binding.^24^ Deletion of the WSXWS conserved motif of CRM‐1 does not affect TPO‐binding capacity.^23^ Instead, the WSXWS motif present at the base of the extracellular domain is known to stabilize the ligand‐binding conformation of type I receptors.^26^, ^27^ Analysis of selected mutations from congenital amegakaryocytic thrombocytopenia (CAMT) patients and structure‐guided mutagenesis revealed that F45, L103, R102 and F104 are potential ligand‐binding sites.^23^, ^28^ Among these, TpoR R102P found in CAMT patients is restricted in the endoplasmic reticulum (ER). Interestingly, TpoR R102P is rescued for traffic to the cell surface and subsequent activation by TpoR agonist eltrombopag^29^ when coexpressed with CALR exon 9 mutants (found in myeloproliferative neoplasm). Recently, a novel TpoR mutation (TpoR R464G) has been detected in patients diagnosed with CAMT.^30^ TpoR R464G could not be detected on the surface of platelets in these patients although low levels of surface expression coupled with limited activation by TPO were observed when expressed in Ba/F3 and UT‐7 cell lines. However, unlike TpoR R102P, co‐expression of CALR mutant could not rescue the traffic defect of TpoR R464G. The exact mechanism of action of CALR mutants on these traffic‐deficient TpoR, however, remains elusive.

The transmembrane and juxtamembrane domain (22aa) folds into an α‐helix and acts as the control centre for dimerization and activation of the receptor. This region may also exist in different dimeric‐conformational orientations.^31^ Experiments by fusing Put 3 coiled‐coil domains to the transmembrane region of the TpoR and engineering the junction of dimeric coiled‐coil and TpoR showed that it could signal from 6 different orientations. The extent of signalling may differ, as evidenced by the differences in the proliferation of Ba/F3 and UT‐7 cell lines expressing these constructs.^31^ Although the transmembrane region is composed of a relatively short stretch of amino acids when compared to the full receptor, this region plays a crucial role in halting ligand‐independent activation of TpoR. Specifically, H499 and RW_515_QFP are two essential motifs for the prevention of ligand‐independent activation of TpoR.^32^, ^33^ Incidentally, eltrombopag, an agonist of TpoR which binds at position H499, is used to treat thrombocytopenia.^32^, ^34^, ^35^ W515, part of the RW_515_QFP motif located at the juxtamembrane region, is responsible for maintaining TpoR in an inactive state in the absence of its ligand. Hence, mutation at W515 to any residue except proline and cysteine (W515P and W515C) results in constitutive activation of TpoR.^36^ In fact, myeloproliferative neoplasm (MPN) patients with essential thrombocythemia (ET) and primary myelofibrosis (PMF) were found to harbour activating transmembrane domain mutations W515L/K/R/A, S505N and V501A.^37^ Of these, S505 and V501 appear at the dimeric interphase and maintain the inactive conformation of the receptor. Recently, saturation mutagenesis of the transmembrane domain revealed that second site mutations in the same domain modulated the effects of these driver mutations. For example, R514K enhanced the ligand‐independent activation of S505N.^38^ Moreover, aberrant activation of the TpoR extracellular domain by oncogenic mutations S505N and W515A/L/K was found to depend upon W491 residue of the extracellular domain.^39^ Taken together, amino acids at dimeric interface (V501, S505) and juxtamembrane region (W515, L498) prevent ligand‐independent activation of the receptor.^38^, ^39^

Of note, the transmembrane domain of murine TpoR has higher propensity for dimerization in comparison with human TpoR. This has been attributed to the absence of the H499 residue in murine TpoR that results in an uninterrupted helical conformation of the transmembrane domain favouring the activation of murine TpoR.^32^ Indeed, such a scenario is observed in v‐MPL, an oncoprotein present in murine myeloproliferative leukaemia virus (MPLV).^3^ v‐MPL was observed to lack most of the extracellular domain of murine TpoR that possibly blocks activation of the receptor in the absence of its ligand. Instead, the oncoprotein contained the transmembrane and intracellular domain of murine TpoR fused to a 100 amino acid segment of Friend murine leukaemia virus envelope protein, resulting in transformation of haematopoietic progenitors, autonomous cell growth and robust myeloproliferation.

The cytoplasmic domain is the signalling hub of the receptor. It consists of 122aa and has many unique features such as conserved box 1, box 2 and the presence of 5 tyrosine residues (Y521, Y542, Y591, Y626 and Y631, for the human TpoR). Boxes 1 and 2 are essential for recruiting JAK2.^40^, ^41^ Because TpoR does not exhibit intrinsic tyrosine kinase activity, the receptor depends on cytoplasmic non‐receptor tyrosine kinases such as JAK2 and TYK2 for triggering activation and signal transduction. Following ligand binding, the cytoplasmic domain initiates the signalling cascade through conformational changes in the receptor. Ligand‐dependent signalling induces strong dimerization of the receptor in the presence of JAK2.^42^ JAK2 phosphorylates TpoR on Y626 and Y631 and then cross phosphorylates each other.^43^, ^44^, ^45^ Consequently, STAT 1/3/5 binds to phosphorylated receptor through SH‐2 domains and JAK2 further phosphorylates STATs.^46^, ^47^, ^48^ Phosphorylation of STATs leads to their dimerization. Dimerized STATs finally enter the nucleus to carry out TpoR‐specific transcription. Moreover, JAKs have also been shown to activate other pathways such as MAPK^49^ and PI3K^50^ pathways.

## 2 RECEPTOR DIMERIZATION

Previously, it was thought that TpoR exists on the surface as preformed dimers or in a monomer‐dimer equilibrium. Biophysical studies using the transmembrane and juxtamembrane domains indicated that unstimulated human TpoR might be monomeric, while the murine TpoR might exist at least partially as preformed dimers.^32^ These differences between the murine and human TpoR were attributed to the H499 residue in the transmembrane domain that is unique to the human receptor.^32^, ^51^ Indeed, H499 interrupts the alpha helix that might be required for preformed dimerization in an inactive orientation.^32^ Dimerization of TpoR has also been detected in cells using various methods such as cysteine cross‐linking assay, TOXCAT or FRET‐based protein‐protein interaction assays.^31^, ^52^ However, these approaches may give rise to a false interpretation of preformed dimers as these techniques can detect weak interactions between the receptor monomers.^52^ Besides, elevated cell surface expression of TpoR and ensuing crowding and weak interactions of monomers cannot be ruled out.^42^ Recently, utilizing physiological expression and single‐molecule co‐locomotion imaging of post‐translationally labelled full‐length surface monomers, it has been shown that class I cytokine receptors TpoR, EpoR and GHR predominantly exist on the surface as monomers which form stable signal‐transducing dimers only when bound to their ligands.^42^ It is important to note that using a similar technique, low‐affinity IL6‐R ligand (C7 and A1 IL6 engineered variants) displayed low to no dimerization but carried out strong STAT5 activation indicating that signalling and dimerization may be uncoupled at least for low‐affinity IL6‐R ligands.^53^

When bound by its ligand, stable dimerization of TpoR occurs aided by the dimerization of the JAK2 pseudokinase (PK) domain. This paves the way for the activation of the C‐terminal tyrosine kinase domain of JAK2. Deletion of the PK domain leads to reduced stability of active TpoR dimers, while the absence of JAK2 causes lower levels of intrinsic dimerization of TpoR.^42^ A stabilizing interaction between JAK2 PK domains has been hypothesized by Wilmes et al. to be important for the dimerization of cytokine receptors. Along these lines, the FERM domain mutation of JAK2 (JAK2 L224E), which inhibits the PK‐PK interaction, significantly inhibits TpoR dimerization even in the context of the activating PK domain mutation JAK2 V617F (driver mutation in MPN). Therefore, the extent of TpoR dimerization and signalling is determined by FERM and PK domains of JAK2 as shown by L224E and V617F mutations, respectively.^42^ Furthermore, experiments with TpoR W515L and JAK2 V617F have provided insight into factors affecting ligand‐independent dimerization of TpoR. W515L has been shown to induce strong dimerization in the presence of JAK2, only transient weak dimerization in the absence of JAK2, while minor dimerization has been observed with TYK2. JAK2 V617F drives effective ligand‐independent dimerization of TpoR and EpoR,^54^ while substantially less dimerization was observed for GHR. Similar to TpoR, and for all the three IL‐4 receptor complexes, IL‐4:IL‐4Rα/IL‐13Rα1 (type 2 complex), IL‐13:IL‐4Rα/IL‐13Rα1 (type 2 complex) and IL‐4:IL‐4Rα/IL‐2Rγ (type 1 complex); fluorescence cross‐correlation spectroscopy, as well as intramembrane dissociation constants, indicated the formation of short‐lived transient dimers which are incapable of triggering signalling in the absence of the ligand.^55^ Taken together, TpoR appears to exist as signalling competent dimers only in the presence of its ligand. Ligand‐independent TpoR dimers are stabilized by specific mutations in JAK2 (JAK2 V617F) that enhances PK‐PK interactions of adjacent JAKs or TpoR juxtamembrane mutations (TpoR W515) that relieve conformational inhibition.

## 3 REGULATION OF TPOR SIGNALLING

Ligand stimulation leads to stable dimerization of TpoR. Dimerization triggers downstream signalling molecules such as STATs causing TpoR‐specific transcription that maintains HSC population^11^, ^12^, ^15^, ^16^ as well as platelet production.^13^, ^17^ Having such an important role in haematopoiesis, TpoR signalling must be tightly regulated through multiple mechanisms. TpoR signalling is known to be regulated by two means. The first involves activation of the negative regulators of TpoR signalling cascade such as suppressor of cytokine signalling (SOCS), and ^56^, ^57^ PIAS^58^, ^59^‐PIAS inhibits STAT DNA‐binding activity, LYN^60^ and LNK.^61^ Further details may be found in the reviews.^62^, ^63^, ^64^ The second method is by internalization of the active receptor complexes followed by degradation or recycling of the receptor to the surface resulting in attenuation of downstream signalling.^65^ TpoR internalization has been followed by co‐staining the cells for transferrin endocytosis.^66^ Such experiments showed that endocytosed TpoR accumulates in early‐endosomal vesicles. TpoR endocytosis is regulated by dynamin as chemical inhibition using Dynasore‐blocked TpoR internalization.^29^ Importantly, *Dnm2* knock‐out mice showed pronounced macrothrombocytopenia with impaired TpoR endocytosis.^67^ Impaired endocytosis was accompanied by sustained TpoR signalling and JAK2 phosphorylation. Therefore, internalization of active TpoR complexes is essential to attenuate TpoR signalling. Additionally, the phenotype of macrothrombocytopenia appears to be paradoxical, suggesting that excessive signalling by TpoR may have negative consequences.^66^, ^68^ TpoR cytoplasmic domain associates with AP2 to induce TPO‐stimulated and clathrin‐mediated endocytosis of the receptor.^65^ Internalization of TpoR is controlled by two intracellular motifs Y626 (fourth cytoplasmic tyrosine residue) and box 2 region (L_567_ L_568_ E_569_I_570_ L_571_) containing dileucine motifs (L_567_ L_568_ and I_570_ L_571_). However, box 2 exhibits internalization independent of JAK2 activation.^69^ This is consistent with the study done by Royer et al. concluding that only box 1 and sequence between box 1 and box 2 (Q_532_Y_533_L_534_ in murine homologue) are required by FERM domain of JAK2 to enhance cell surface expression as well as internalization of TpoR.^70^ When stimulated with TPO, TpoR is ubiquitinated at K_553_ and K_573_ resulting in degradation of the receptor.^71^ This is mediated by E3 ubiquitin ligases such as CBL. Crucially, CBL mutations are frequently detected in MDS/MPN.^72^ It may be noted that G‐CSFR continues signalling even after internalization in early endosomes.^73^ As observed in the case of K5R mutant of G‐CSFR where all the cytoplasmic lysines are mutated to arginine, STAT5 and ERK activation increases after internalization to early endosomes without being localized into late endosomes and lysosomes. Similarly, engineered high‐affinity IL6 ligands (HyIL6) co‐localized in early‐endosomal compartment while low‐affinity ligands displayed little or no colocalization in the same compartment. This correlated with robust STAT1/3 activation by HyIL6 as compared to low‐affinity ligands.^53^ Recent data have revealed that MPN‐associated CALR mutants induced early‐endosomal localization of TpoR.^29^ Whether early‐endosomal localization contributes towards increased signal amplitude remains unexplored for TpoR‐mutant CALR complexes.

Platelet surface TpoR acts as a rheostat by regulating the availability of free circulating TPO in the blood. Similar to megakaryocytes, platelet TpoR binds to serum TPO resulting in the endocytosis of the complex. Endocytosis is mediated by Dynamins as evidenced by impaired TPO‐induced TpoR endocytosis resulting in increased serum TPO levels in *Dnm2* ^−/−^ mice.^67^ Additionally, mislocalized early‐endosomal markers (EEA1) and abnormal clustering of clathrin away from the plasma membrane were observed in *Dnm2* ^−/−^ megakaryocytes. Together, these data indicate a clathrin and dynamin‐dependent endocytosis of platelet surface TpoR. Platelets also show the presence of recycling endosomes^74^ along with a characteristic TpoR surface recovery kinetics following TPO stimulation.^67^ However, very little information is available regarding the other molecular components involved in endocytosis of the TPO‐TpoR complex and recycling/degradation of TpoR, which may be different in HSCs and early MK progenitors. Importantly, TpoR expression correlates with the number of hematopoietic stem cells,^12^ megakaryocyte progenitors, megakaryocytes and platelets.^75^, ^76^ Mice lacking TPO or TpoR are severely thrombocytopenic and deficient in megakaryocytes and their progenitors.^75^ When TpoR is expressed in progenitors but not in megakaryocytes and platelets, platelet numbers increase due to lack of internalization and clearance of TPO from circulation.^76^ Additionally, TPO has been shown to prime HSCs towards the megakaryocyte lineage.^77^ This explains the paradoxical thrombocytosis observed in *mpl* ^−/−^ mice engineered to express low levels of TpoR wherein excess serum TPO enhances megakaryopoiesis.^78^ Similarly, two partially traffic‐deficient TpoR, *viz*. TpoR K39N and P106L mutants, result in hereditary thrombocytosis due to the presence of excess TPO in circulation, which stimulates megakaryocyte progenitor proliferation.^79^ A recent study by Favale et al. on TpoR P106L showed low surface expression of the receptor in megakaryocyte progenitors and UT‐7 cells.^79^ TpoR P106L accumulates in the ER and can traffic to the surface, possibly through a Golgi‐independent route.^79^ Low TpoR P106L activity is correlated with low surface expression and an internalization defect. Of note, the region between R102 and P106 is important for cell surface expression of TpoR as well as its ligand‐binding activity. While R102P is blocked in the ER and is unresponsive to TPO, P106L shows the partial response to the ligand.^79^

## 4 TPOR ACTIVATION BY AGONISTS

TpoR agonists have been designed to tune TpoR signalling by either decreasing the distance between the monomers or changing the dimeric‐conformational interface or dimeric topology, making the receptor active to various extents. These include eltrombopag, romiplostim and diabodies. Eltrombopag was first identified in a high‐throughput screen of small molecule compounds capable of activating STAT in Ba/F3 cells expressing TpoR.^80^ Crucially, eltrombopag was found not to compete with Tpo for binding to TpoR. Instead and as previously described, eltrombopag was observed to bind to H499 residue of human TpoR resulting in effective dimerization and activation of the receptor.^32^ The dependency on H499 residue also marks a crucial difference between murine and human TpoR whereby only human TpoR containing H499 is activated by eltrombopag. Moreover, asparagine‐scanning mutagenesis of the transmembrane domain of murine TpoR revealed that mutation at several residues was capable of activating the receptor. However, only S505N was found to activate the human TpoR. This difference was attributed to the presence of H499 which induced a local non‐helical conformational around H499. Binding of eltrombopag resulted in induction of helical conformation around H499 favouring dimerization and activation of the receptor. Similarly, a peptide screening assay to identify peptides that bound to TpoR with high affinity leads to the identification of a 14 amino acid peptide (IEGPTLRQWLAARA). Dimerization of this peptide by a carboxyl‐terminal linkage to a lysine branch yielded peptides having almost equal affinity to TpoR as the natural ligand TPO.^81^ This knowledge was later used to develop Romiplostim which is a recombinant fusion protein consisting of two identical subunits, each having IgG1 Fc domain linked to two TpoR binding domains. Romiplostim was found to bind to the TpoR at regions similar to its natural ligand TPO. Moreover, romiplostim induced strong internalization of the receptor along with the activation of STAT, Akt and Erk signalling.^82^ Presently, both eltrombopag and romiplostim are used in the treatment of idiopathic thrombocytopenic purpura (ITP).

Diabodies on the other hand are dimeric, bivalent antibody fragments constructed by joining heavy chain variable region and light chain variable region genes with five‐mer linker sequences. They are designed to bind to the extracellular domain of the receptors. Due to their close binding sites, diabodies induce effective dimerization and activation of the receptor. The binding epitope of different diabodies may differ. This translates into different signalling output as well as varying levels of competition with the natural ligand. In a recent study, Cui et al.^83^ tested the binding of three diabodies AK111, AK113 and AK119 to the TpoR and their effects on receptor dimerization, activation and signalling. While AK111 reduced TPO binding up to 100%, AK119 showed robust dimerization and activation of the downstream signalling pathways. Thus, diabodies can effectively induce graded TpoR activation and signalling. It is possible that diabodies could also induce different levels of receptor internalization which could translate into different degrees of activation of the receptor. Such a possibility needs to be explored in future.

Another method to fine‐tune the dimeric topology of the receptors involves the use of designed ankyrin repeat protein (DARPin) scaffolds that bind with high affinity to receptors of interest. Such a study on EpoR revealed that topological orientation of the extracellular domain could alter the proximity, orientation and topology of the associated JAKs leading to changes in signalling amplitude.^84^ Indeed, DARPins may be useful in deciphering the topological orientations of TpoR extracellular domain relative to the intracellular signalling pathways especially in terms of the various activating mutations of the transmembrane domain of TpoR. The importance of topological orientation is highlighted by a recent study on TpoR R464G. The TpoR R464G was observed to be unresponsive to TPO or eltrombopag.^30^ However, upon co‐expression of CALR del52, TpoR R464G showed selective activation with eltrombopag alone. It is possible that R464G mutation locks TpoR in an inactive topological orientation which is relieved upon binding to CALR del52 making it accessible to eltrombopag.

## 5 TPOR SURFACE LOCALIZATION IS MEDIATED BY JAK2 AND TYK2

TpoR has four sites for N‐glycosylation (N_117_, N_178_, N_298_ and N_358_). While core glycosylation at the four Asn residues occurs in the ER, the addition of mature glycans requires passage through the Golgi. TpoR is expressed on the surface as mature Golgi processed, as well as immature glycosylated forms. JAK2 and TYK2 regulate the ratio of mature to immature form and promote surface localization of the mature Golgi processed form of the receptor by enhancing the recycling and stability of the receptor.^70^ Additionally, JAK2 and TYK2 increase the total protein level of TpoR by protecting against proteasomal degradation. Mutant JAK2 V617F exhibits down‐modulation of surface TpoR and total TpoR levels.^66^, ^85^ The decrease in cell surface expression of TpoR in the presence of JAK2 V617F mutant is accompanied by impaired recycling of TpoR to the surface. Furthermore, the internalization of TpoR in JAK2 V617F mutant is much more pronounced, as compared to JAK2 WT. JAK2 V617F mediates down‐modulation of TpoR through enhanced ubiquitination and degradation of the receptor. Hence, inhibitors of JAK2 and proteasomal degradation have been shown to restore surface expression of TpoR in JAK2 V617F mutant cells. Insight into the mechanism of JAK2/TYK2‐mediated enhanced half‐life of receptors is obtained from the type 1 IFN receptor complex. IFNAR1 subunit internalization is uniquely regulated by TYK2. In this receptor, TYK2 masks the internalization motif, thereby inhibiting ligand or ubiquitination independent internalization.^86^ In the absence of the ligand, TYK2 prevents the interaction between the internalization motif (Y466) on IFNAR1 and AP50 (subunit of AP2). These observations show that the masking effect of TYK2 reduces the basal internalization rate, thereby increasing the half‐life of IFNAR1.^86^

## 6 GOLGI‐INDEPENDENT TRAFFIC OF TPOR

**FIGURE 2:**
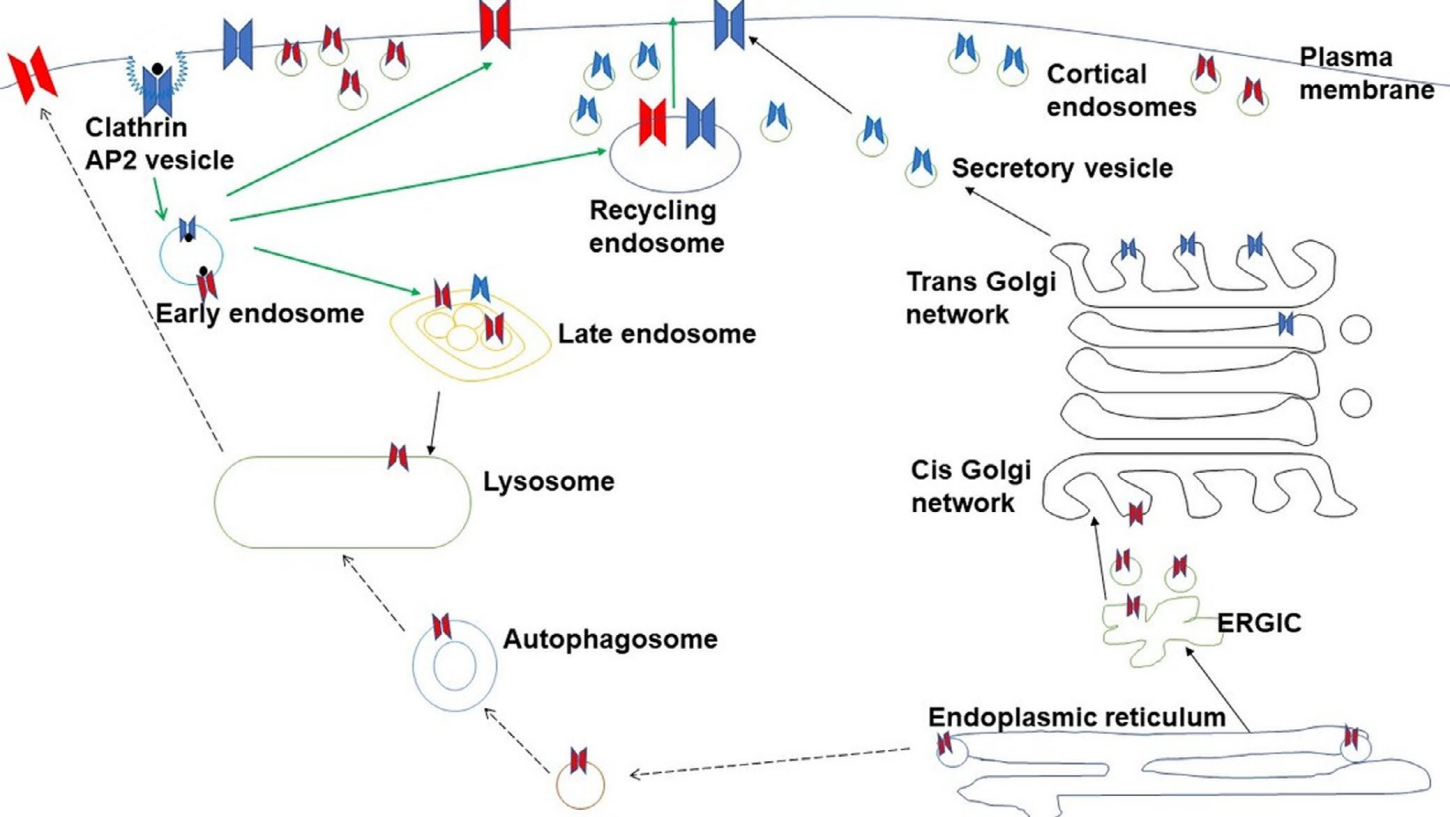
TpoR traffic routes. The classical Golgi‐dependent (solid lines), autophagosome‐lysosome dependent (dashed lines) traffic routes and the endocytic pathway (in green arrow) are depicted. Glycosylation status of TpoR—immature (in red) and mature (in blue) are indicated throughout the TpoR traffic routes

TpoR utilizes both Golgi‐dependent and Golgi‐independent routes for traffic to the cell surface (Figure 2). The Golgi‐dependent route of TpoR traffic marks exits through the ER‐Golgi to the cell membrane (anterograde secretion pathway) and is used by complex glycosylated TpoR. The Golgi‐independent pathway is utilized by TpoR containing immature glycosylation and is believed to be processed through autophagosomes to the cell surface.^87^ A recent study published by Cleyrat et al.^87^ indicates that TpoR colocalizes with low pH and autophagic multivesicular body markers LC3, LAMP1 and Rab11 in K562 and HEL cells. Additionally, autophagy inducers (Rapamycin and GRASP 55) led to increased immature form of TpoR, suggesting that a fraction of TpoR bypasses the Golgi and utilizes autophagy‐dependent unconventional secretory pathway for traffic to the surface. On the other hand, addition of calcium ionophores (PMA and A23187) caused the reappearance of mature TpoR on the surface presumably due to accelerated fusion of vesicles. Therefore, both Golgi‐dependent and autophagy‐dependent traffics of TpoR may co‐exist in cells. Interestingly, analysis of the ligand‐induced recovery of the receptor revealed rapid recycling of immature TpoR as compared to the mature form of TpoR. The authors suggested that a pool of immature TpoR was present in vesicles near the cell membrane that had bypassed the Golgi and trafficked through LAMP1 ^+^ and/or LC3^+^ vesicles in K562 and HEL cell lines expressing TpoR. Like TpoR, IL‐4R subunits localize in early and recycling endosomes, with very low localization in late endosomes and lysosomes in the absence of ligand. This shows that a significant fraction of receptors such as TpoR and IL‐4R remain in cortical endosomes, which contribute towards the rapid recycling of receptors.^55^ Association of TpoR with JAK2 is essential for the presentation of mature TpoR to the surface through an anterograde secretion pathway.^87^ It is known that JAK2 and TYK2 increase the half‐life of the mature form of TpoR.^70^ However, mutant JAK2 V617F coexpressed with TpoR in Ba/F3 cell line has been shown to increase the half‐life of the immature form of TpoR rather than mature TpoR.^66^ Although surface expression of immature TpoR appears to be correlated with Golgi‐independent traffic, it remains to be verified in the case of TpoR associated with CALR exon 9 mutants and JAK2 V617F. Further details of surface expression, N‐glycosylation status and traffic routes for the various mutants are provided in Table 1.

**TABLE 1:** Effects of the expression of WT and mutant TpoR, JAK2 and CALR on the cell surface expression of TpoR

Mutations	TpoR surface expression	TpoR glycosylation	Pre‐dominant pathway for traffic	References
JAK2 WT	High (++++)	Mature	Secretory	Cleyrat et al. (2014)^87^; Royer et al. (2005)^70^; Pecquet et al. (2012)^66^
TYK2 WT	Moderate (++)	Mature	Secretory	Royer et al. (2005)^70^
JAK2 V617F	Moderate (++)	Immature	Lysosomal	Cleyrat et al. (2014)^87^; Pecquet et al. (2012)^66^
CALR WT	High like TpoR WT (+++)	Mature	Secretory	Pecquet et al. (2019)^29^
CALR del52	Moderate (++)	Immature	Secretory	Pecquet et al. (2019)^29^
CALR ins5	Moderate (++)	Immature	Secretory	Pecquet et al. (2019)^29^
TpoR K39N	Low (+)	Mature +Immature	Unknown	Pecquet et al. (2019)^29^; Moliterno et al. (2004)^107^
TpoR R102P	Absent (‐)	N/A	N/A	Varghese et al. (2014)^25^
TpoR R102C	Absent (‐)	N/A	N/A	Ballmaier et al. (2001) ^108^; Varghese et al. (2014)^25^
TpoR F104S	High like TpoR WT (+++)	Mature	Secretory	Stockklausner et al. (2015) ^109^ ;Varghese et al. (2014)^25^
TpoR P106L	Low (+)	Immature	Lysosomal	Stockklausner et al., 2015 ^109^; Favale et al. (2016)^79^
TpoR D128Y	High like TpoR WT (+++)	Unknown	Unknown	Varghese et al. (2014)^25^
TpoR P136L	Moderate (++)	Unknown	Unknown	Varghese et al. (2014)^25^
TpoR P267T (murine)	Moderate (++)	Unknown	Unknown	Varghese et al. (2014)^25^
TpoR G434R (murine)	Low (+)	Unknown	Unknown	Varghese et al. (2014)^25^
TpoR G509N	Low (+)	Immature	Unknown	Pecquet et al. (2019)^29^; Leroy et al.^32^
TpoRCysless (cysteine mutants‐folding deficient)	Absent (‐)	N/A	N/A	Pecquet et al. (2019)^29^
TpoR D1D2	Absent (‐)	N/A	N/A	Pecquet et al. (2019)^29^
TpoR box 1/box 2 mutant	Absent (‐)	N/A	N/A	Royer et al. (2005)^70^
TpoR R464G	Low (+)	Immature	Unknown	Basso‐Valentina et al. (2021)^30^

Note

Effects of the expression of WT and mutant TpoR, JAK2 and CALR on the cell surface expression and glycosylation status of TpoR has been indicated. The major route (Glogi dependent/Lysosomal) for TpoR traffic in the various conditions has been shown. N/A indicated not applicable.

## 7 CALRETICULIN MUTATIONS IN MPN

CALR is an ER‐resident chaperone with three distinct functional domains.^88^ The N‐terminal domain contains glycan‐dependent and glycan‐independent polypeptide‐binding sites essential for its chaperone activity. The high‐affinity Ca^2+^‐binding site containing proline‐rich P‐domain interacts with the thiol oxido‐reductase Erp57 and is involved in glycan‐independent chaperone activity. The acidic C‐terminal domain contains multiple high capacity, low‐affinity Ca^2+^ binding sites that regulate ER Ca^2+^ buffering and homeostasis. The C‐terminus ends with the KDEL retrieval sequence for retrograde transport of CALR from the Golgi and ER‐Golgi intermediary complex (ERGIC) to the ER lumen. Functionally, CALR in concert with calnexin ensures proper folding of mono‐glycosylated high mannose containing glycoproteins.^89^ *CALR* mutations were first identified in patients with MPN namely essential thrombocythemia and primary myelofibrosis in 2013.^90^, ^91^ In these patients, *CALR* mutations resulted in megakaryocyte hyperplasia and myeloproliferation. The exon‐intron organization of the *CALR* gene shows that the N‐terminal domain is encoded by exons 1–4, and P‐domain is encoded by exons 5– 7 whereas exons 8 and 9 encode the C‐terminal domain. Strikingly, MPN‐associated +1 frameshift mutations cluster in the exon 9 of *CALR*. The two most common mutations in *CALR* include *CALR del52* (type I) and *CALR ins5* (type II). These mutations differ in the length of the WT C‐terminus tail that is retained in the mutant protein with *CALR ins5* retaining a portion of the WT exon 9 sequence. Further details of the various genetic alterations associated with MPN may be found in the review.^92^ *CALR* mutations result in partial (*CALR ins5*) or near‐complete (*CALR del52*) elimination of the acidic C‐terminal domain along with elimination of the KDEL retrograde transport signal. The novel tail is rich in positively charged amino acids Met and Arg. It has been suggested that alterations in ER Ca^2+^ buffering capacity of the mutants along with defective interaction with store‐operated calcium entry (SOCE) proteins result in mobilization of ER Ca^2+^.^93^, ^94^ Interestingly, CALR mutants selectively activated TpoR and to a weaker extent G‐CSFR.^95^ Furthermore, shRNA‐mediated knock‐down of JAK2 or TpoR resulted in decreased number of CD34^+^ cell‐derived TPO‐independent CFU‐Mk from *CALR del52* and *CALR ins5* harbouring ET patients.^95^ These data point towards a mutant CALR‐TpoR‐JAK2 axis‐driven myeloproliferation. Moreover, it explains the phenotypic observation of dysregulated megakaryopoiesis in mutant CALR‐driven MPN.

## 8 CALRETICULIN MUTATIONS AND TRAFFIC OF TPOR

CALR mutations associated with exon 9 of CALR resulted in a positively charged tail devoid of the ER retention signal KDEL. Therefore, it was speculated that the mutants might exit ER in high numbers. Indeed, data from multiple groups have conclusively shown that CALR mutants follow the classical Golgi‐dependent secretory pathway.^29^, ^96^, ^97^ The mutants have been observed in the ER, Golgi, ER‐Golgi intermediary compartment (ERGIC), endosomal vesicles, plasma membrane and even in the nucleus.^29^, ^95^, ^98^ Moreover, recent data have indicated that the CALR mutants are heavily secreted and not only can modulate immune response^96^ but may also act as trans‐acting paracrine factors for TpoR stimulation.^99^ Recent publications have indicated that MPN‐associated CALR mutants downmodulate the expression of TpoR.^95^, ^100^ Mutant CALRs show multiple effects on TpoR structure and function. Interaction of TpoR with CALR mutants begins inside the ER lumen where the lectin‐binding domain of mutant CALRs associates with high affinity to (GlcNAc)_2_(Man)_9_Glc residue on TpoR.^29^ Therefore, mutant CALRs specifically co‐immunoprecipitated with immature high mannose containing TpoR.^95^ The mutant CALR‐TpoR interaction is possibly retained as the complex exits ER and traverses through the Golgi to the cell surface. This has been attributed to the stable binding of mutant CALR to the immature glycan, especially on N117 of TpoR. The complex between mutant CALR and TpoR prevents further processing of N117‐linked sugars during passage through the Golgi.^29^ While this has been observed in insect cells coexpressing mutant CALR and the soluble TpoR extracellular domain, whether such a scenario is indeed responsible for the surface appearance of immature glycosylated TpoR remains to be explored. Oligomerization of mutant CALRs aided by its novel C‐terminal tail induces signalling competent dimerization of cell surface TpoR.^101^ Hence, the TpoR‐mutant CALR complex induces JAK2‐STAT1/3/5 signalling. Moreover, endosomal localization of active TpoR complexes has also been detected in the presence of CALR mutants.^29^ Interestingly, inhibition of TpoR endocytosis increased mutant CALR‐dependent STAT5 signalling.^29^ Thus, it appears that surface expression of the TpoR‐mutant CALR complex is necessary for TpoR signalling.^29^, ^97^ Mutant CALR induced dimerization of TpoR in the presence of JAK2 but failed to do so for EpoR, indicating specificity for the association between mutant CALR and TpoR.^95^, ^102^ Interaction between CALR mutant and TpoR is primarily dependent upon N‐glycosylation (especially N117 residue). The N117 residue is also conserved in murine TpoR. A hydrophobic patch present at the extracellular domain of TpoR was also found to regulate mutant CALR‐dependent TpoR signalling.^29^ Alanine mutation in the hydrophobic patch (TpoR 8A) led to diminished signalling activity and thermal stability of TpoR in the presence of CALR mutants but retained interaction with mutant CALRs. Mutant CALRs have been found to destabilize the protein‐protein interaction characteristic of CALR WT such as formation of the peptide loading complex and interactions with Erp57.^103^, ^104^ Yet, a stabilizing effect of mutant CALRs especially CALR del52 has been observed on TpoR.^29^ CALR del52 increased the thermal stability of TpoR both in cell lines and in primary *Calr del52* knock‐in mouse platelets. This has a direct consequence on the surface expression of traffic‐deficient TpoR mutants. CALR del52 induced traffic and surface expression of TpoR R102P and enhanced surface expression of TpoR P106L. Thus, a chaperone‐like effect of mutant CALRs was observed on these traffic‐deficient TpoR mutants.

## 9 PERSPECTIVES

Defects in TpoR traffic are associated with multiple pathological conditions. Yet, the exact mechanisms of TpoR traffic and sorting post‐receptor endocytosis remain unknown. Recent reports on fibrinogen endocytosis in platelets have implicated Arf6 (small Ras‐like GTP‐binding protein) and VAMP‐3 (v‐SNARE) proteins in the process.^74^, ^105^ It remains to be seen whether these effectors also modulate endocytosis of TpoR. Although the conventional anterograde transport of TpoR has been widely studied, we are just beginning to appreciate the unconventional autophagosome‐lysosomal route. It is not clear how much each of these routes contributes towards surface TpoR expression in HSCs, progenitors and platelets. Moreover, how these routes affect the pathophysiology of TpoR in relation to TpoR/JAK2/CALR mutations needs to be explored. For example, the unconventional autophagosome‐lysosomal traffic of TpoR could be detected in cells with JAK2 V617F or TpoR P106L.^66^, ^79^ However, it remains to be established whether blocking the unconventional traffic of TpoR affects the disease pathology. Unconventional traffic that bypasses Golgi would necessitate changes in TpoR N‐glycosylation. Of note, mutations targeting individual Asn residues responsible for N‐glycosylation of TpoR showed little effect on its surface expression and response to TPO.^106^ However, combinatorial mutations did indeed decrease surface expression and TpoR signalling. Nevertheless, we do not understand whether the N‐glycosylation status affects receptor internalization and membrane dynamics. Answers to these questions will serve to better understand paradoxical thrombocytosis and aid in the development of effective TpoR agonists/antagonists.

## CONFLICT OF INTEREST

The authors declare no conflict of interest.

## AUTHOR CONTRIBUTIONS

**Anita Roy:** Data curation (lead); supervision (lead); writing—original draft (lead); writing—review and editing (lead). **Saurabh Shrivastva:** Data curation (equal); writing—original draft (equal); writing—review and editing (equal). **Saadia Naseer:** Data curation (equal); writing—original draft (equal); writing—review and editing (equal).
